# Thalamus and focal to bilateral seizures: A multiscale cognitive imaging study

**DOI:** 10.1212/WNL.0000000000010645

**Published:** 2020-10-27

**Authors:** Lorenzo Caciagli, Luke A. Allen, Xiaosong He, Karin Trimmel, Sjoerd B. Vos, Maria Centeno, Marian Galovic, Meneka K. Sidhu, Pamela J. Thompson, Danielle S. Bassett, Gavin P. Winston, John S. Duncan, Matthias J. Koepp, Michael R. Sperling

**Affiliations:** From the Department of Clinical and Experimental Epilepsy (L.C., L.A.A., K.T., S.B.V., M.C., M.G., M.K.S., P.J.T., G.P.W., J.S.D., M.J.K.) and Neuroradiological Academic Unit (S.B.V.), UCL Queen Square Institute of Neurology, London; MRI Unit (L.C., L.A.A., K.T., S.B.V., M.C., M.G., M.K.S., P.J.T., G.P.W., J.S.D., M.J.K.), Epilepsy Society, Chalfont St Peter, Buckinghamshire, UK; Departments of Bioengineering (L.C., X.H., D.S.B.), Physics and Astronomy (D.S.B.), Electrical and Systems Engineering (D.S.B.), Neurology (D.S.B.), and Psychiatry (D.S.B.), University of Pennsylvania, Philadelphia; Department of Neurology (K.T.), Medical University of Vienna, Austria; Centre for Medical Image Computing (S.B.V.), University College London, UK; Department of Neurology (M.G.), University Hospital Zurich, Switzerland; Santa Fe Institute (D.S.B.), NM; Department of Medicine, Division of Neurology (G.P.W.), Queen's University, Kingston, Canada; and Department of Neurology (M.R.S.), Thomas Jefferson University, Philadelphia, PA.

## Abstract

**Objective:**

To investigate the functional correlates of recurrent secondarily generalized seizures in temporal lobe epilepsy (TLE) using task-based fMRI as a framework to test for epilepsy-specific network rearrangements. Because the thalamus modulates propagation of temporal lobe onset seizures and promotes cortical synchronization during cognition, we hypothesized that occurrence of secondarily generalized seizures, i.e., focal to bilateral tonic-clonic seizures (FBTCS), would relate to thalamic dysfunction, altered connectivity, and whole-brain network centrality.

**Methods:**

FBTCS occur in a third of patients with TLE and are a major determinant of disease severity. In this cross-sectional study, we analyzed 113 patients with drug-resistant TLE (55 left/58 right), who performed a verbal fluency fMRI task that elicited robust thalamic activation. Thirty-three patients (29%) had experienced at least one FBTCS in the year preceding the investigation. We compared patients with TLE-FBTCS to those without FBTCS via a multiscale approach, entailing analysis of statistical parametric mapping (SPM) 12–derived measures of activation, task-modulated thalamic functional connectivity (psychophysiologic interaction), and graph-theoretical metrics of centrality.

**Results:**

Individuals with TLE-FBTCS had less task-related activation of bilateral thalamus, with left-sided emphasis, and left hippocampus than those without FBTCS. In TLE-FBTCS, we also found greater task-related thalamotemporal and thalamomotor connectivity, and higher thalamic degree and betweenness centrality. Receiver operating characteristic curves, based on a combined thalamic functional marker, accurately discriminated individuals with and without FBTCS.

**Conclusions:**

In TLE-FBTCS, impaired task-related thalamic recruitment coexists with enhanced thalamotemporal connectivity and whole-brain thalamic network embedding. Altered thalamic functional profiles are proposed as imaging biomarkers of active secondary generalization.

Temporal lobe epilepsy (TLE) is the most common focal epilepsy syndrome in adults. Focal to bilateral tonic-clonic seizures (FBTCS), formerly termed secondarily generalized seizures, affect at least a third of people with TLE,^1^ are a major risk factor for seizure-related injuries and sudden unexpected death in epilepsy (SUDEP),^2,3^ and are a predictor of unfavorable postsurgical outcome.^4^ Why some people experience these seizures while others do not remains poorly understood. Presumably, specific functional and structural rearrangements may underlie the propensity for large-scale propagation of epileptic activity underlying this severe seizure type. Enhancing our understanding of the mechanisms leading to FBTCS may provide insight into much-needed novel therapeutic targets.

In TLE, thalamic atrophy represents the most common extratemporal abnormality^5,6^ and relates to derangements of cortico-subcortical connectivity,^6,7^ with unfavorable implications for postsurgical outcome.^8,9^ Converging evidence indicates that subcortical nuclei, particularly the thalamus, may be involved in the propagation of temporal lobe seizures.^10,11^ Resting-state fMRI analyses detected more widespread thalamocortical abnormalities in patients with TLE and FBTCS compared to those with focal seizures (FS) that do not generalize.^12^ Recent work also identified impairment of thalamotemporal structural connections in TLE-FBTCS.^13^

The thalamus contributes to motor planning, language, and memory by promoting cortical synchronization and facilitating cortico-cortical interplay.^14,15^ Cognitive tasks perturb brain network dynamics and evoke complex changes in interregional interactions,^16^ offering a powerful tool to identify disease-specific network traits^17^ that resting-state analyses may not adequately capture.^18^ In vivo, cognition is probed via task-based fMRI. Verbal fluency tasks assess expressive language and allow ascertaining language lateralization in focal epilepsy.^19^ Typical activation patterns encompass fronto-temporo-parietal cortices, mesiotemporal structures, and, notably, bilateral thalamus, with left-sided emphasis.^20,21^

By challenging robustness of a functional network largely overlapping with the putative epileptogenic network of TLE, fluency-related task fMRI provides a powerful framework for assessing intergroup differences in underlying brain network organization. If the occurrence of FBTCS in TLE is related to abnormal thalamocortical interactions, one may expect to detect abnormal thalamic activation and connectivity with cognitive demand.

In this study we pursued a comprehensive characterization of the functional underpinnings of recurrent secondary generalization in TLE. As distinct from previous investigations, we envisioned the use of task-based fMRI to capture specific, FBTCS-associated rearrangements within networks recruited during linguistic processing. We hypothesized that, compared to TLE-FS, TLE with recent FBTCS would exhibit impaired thalamic activation, altered connectivity between thalamus and key symptomatogenic areas, including mesiotemporal and motor regions, and higher overall thalamic relevance for mediating signals within large-scale networks. To test these hypotheses, we employed a verbal fluency fMRI paradigm and a multiscale approach entailing comparison of TLE-FS and FBTCS across (1) task-related activation, (2) task-modulated changes of thalamic functional connectivity, via a psychophysiologic interaction analysis, and (3) graph-theoretical measures of thalamic centrality. We also linked domains of activation, connectivity, and centrality via a composite thalamic functional marker and investigated its potential to discriminate TLE-FS and FBTCS at the individual level.

## Methods

### Participants

For this cross-sectional investigation, we consecutively recruited 113 patients with drug-resistant TLE (55 left [30 female]; 58 right [43 female]), who underwent presurgical evaluation at the National Hospital for Neurology and Neurosurgery (NHNN), London, United Kingdom, between 2008 and 2013 (table 1). All participants underwent prolonged interictal and ictal scalp video-EEG, confirming and lateralizing the epileptic focus to one temporal lobe, and presurgical 3T MRI, with qualitative assessment and quantification of hippocampal volumetry and T2 relaxometry.^22^ Ipsilateral MRI findings included hippocampal sclerosis (n = 32/29, left TLE [LTLE]/right TLE [RTLE]), dysembryoplastic neuroepithelial tumor (n = 5/8, LTLE/RTLE), cavernoma (n = 4/7, LTLE/RTLE), and normal-appearing MRI (n = 14/14, LTLE/RTLE). Contralateral mesiotemporal structures were normal in all cases. History of affective illness, referring to depressive and anxiety disorders, was recorded as detailed previously.^23^ Additional clinical/demographic details are available in appendix e-1 (doi.org/10.5061/dryad.2bvq83bm8)*.*

**Table 1:**
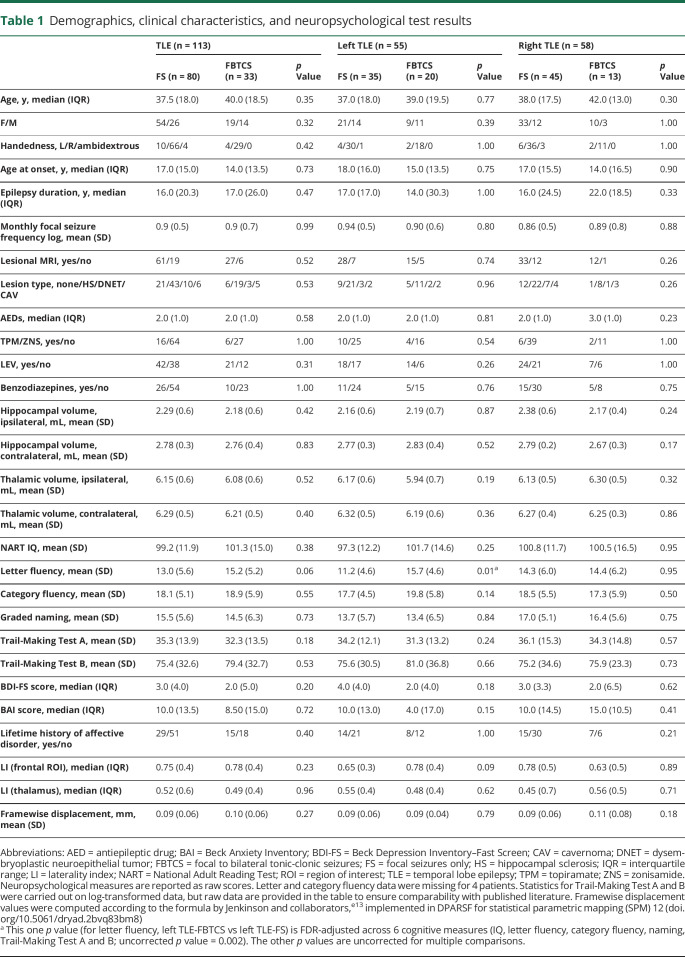
Demographics, clinical characteristics, and neuropsychological test results

Thirty-three patients (29.2%; 20/13, LTLE/RTLE) had experienced at least 1 FBTCS during the year preceding the investigation (median frequency/month: 0.46, interquartile range: 0.83), and were therefore considered as having a current tendency for FBTCS (TLE-FBTCS). This 1-year cutoff for subgroup allocation was envisioned to probe the neural correlates of recent, active secondary generalization, and relies on multiple lines of evidence specifically linking generalized seizures in the last year to SUDEP risk,^2,3^ or recommending assessment of seizure freedom in the last year for clinical outcome classification.^24^ We also conducted post hoc analyses on 3 groups after subdividing the main TLE-FS group into (1) TLE without lifetime history of FBTCS (never FBTCS, n = 38; 14/24, left/right) and (2) TLE with history of remote FBTCS, but none for >1 year before scanning (remote FBTCS, n = 42; 21/21, left/right).

### Standard protocol approvals, registrations, and patient consents

This study was approved by the NHNN and UCL Institute of Neurology Joint Research Ethics Committee. Written informed consent was obtained from all participants.

### Data acquisition and fMRI task specifics

All participants underwent neuropsychological tests measuring intellectual level (IQ), letter and category fluency, and visual confrontation naming. We also evaluated group comparability for processing speed and executive function (table 1 and appendix e-1, doi.org/10.5061/dryad.2bvq83bm8). The Beck Depression Inventory–Fast Screen and Beck Anxiety Inventory measured mood and anxiety. Handedness was determined using the Edinburgh Handedness Inventory. T1-weighted and fMRI data were acquired on a 3T GE (Milwaukee, WI) SignaHDx MRI scanner using previously described protocols (appendix e-1, doi.org/10.5061/dryad.2bvq83bm8).^25^ Automated hippocampal and thalamic volumetric measures were available for all participants. All participants performed a verbal fluency paradigm lasting 5 minutes, consisting of 30-second task blocks requiring participants to covertly generate words beginning with a visually presented letter (A/D/E/S/W; 1 letter per block, 5 blocks in total), alternating with 30-second blocks of crosshair fixation.^26^

### Analysis of clinical and neuropsychological data

For all main analyses on 2 TLE groups (TLE-FBTCS vs TLE-FS), we used the Fisher exact test, 2-sample *t* test, and Mann-Whitney *U* test for categorical, continuous parametric, and nonparametric variables, respectively. Correction for multiple comparisons was attained with the false discovery rate (FDR) procedure. Additional analyses comparing patients with FS and FBTCS were separately carried out for LTLE and RTLE subgroups. Details regarding post hoc analyses on 3 groups (TLE-FBTCS, remote FBTCS, and never FBTCS) are provided at the end of the Methods.

### Imaging data analysis: fMRI activation

We analyzed fMRI data with statistical parametric mapping (SPM) 12 using previously detailed pipelines (appendix e-1, doi.org/10.5061/dryad.2bvq83bm8).^25^ Four participants were excluded owing to corrupted field of view (n = 1) or excessive motion (>|3| mm or |3| degrees overall; n = 3). For each participant, we computed voxel-wise parameter estimates and contrast images for task-related activation, including motion parameters as confounds. At the second level, 1-sample *t* tests assessed fluency-related effects across all participants. Two-sample *t* tests assessed differences between TLE-FBTCS and TLE-FS, with lateralization of the epileptic focus as covariate. Subgroup analyses separately compared LTLE-FS to LTLE-FBTCS and RTLE-FS to RTLE-FBTCS. Age and sex were used as covariates in all group comparisons. Sensitivity analyses entailed repeat group comparisons with letter fluency scores as nuisance regressors. Task effects were thresholded at *p* < 0.05, corrected for multiple comparisons (familywise error rate [FWE]) across the whole brain. In view of our a priori hypotheses, group differences were considered significant at *p* < 0.05, FWE-corrected within a region of interest (ROI) consisting of a 12-mm-diameter sphere (small volume correction [FWE-svc]) centered at the location of the maxima for thalamus, hippocampus, and motor areas (precentral gyrus, supplementary motor area [SMA]).^27^ For completeness, we report whole-brain effects at an exploratory threshold of *p* < 0.005 uncorrected with a 20-voxel minimum cluster-size threshold (*p* < 0.005, k = 20).^28,29^ To convey higher spatial details for our thalamic findings, locations of activation and group difference maxima were related to thalamic subnuclei using the digital version of the Morel stereotactic atlas of the human thalamus.^30^ Hemispheric dominance for frontal and thalamic activation was determined via laterality indices of statistical parametric maps (appendix e-1, doi.org/10.5061/dryad.2bvq83bm8).

### Multiple regression models on thalamic activation

We assessed determinants of task-related thalamic activation via multiple regression models, conducted with *R*-3.4.4. We extracted parameter estimates of thalamic activation from an independent ROI, represented by the ventral anterior nucleus (parvocellular part) of the Morel atlas,^30^ and used the following independent variables: occurrence of FBTCS in the last year, focal seizure frequency (log), sex, handedness, lateralization of the epileptic focus, number of antiepileptic drugs (AEDs), and affective history. For dimensionality reduction, measures of verbal fluency (letter/category fluency) and disease load (age at onset, disease duration) were entered into principal component analyses (PCAs; appendix e-1, doi.org/10.5061/dryad.2bvq83bm8). Both first principal components (“fluency” and “chronicity”) were then implemented as additional regressors.

### Task-related functional connectivity: psychophysiologic interactions (PPIs)

We probed thalamic connectivity with a PPI analysis,^31^ testing whether connection strength between a prespecified seed region and other brain areas was modulated by task execution. Individual fMRI time-series were obtained from the preprocessed images using a 12-mm diameter sphere centered on individual, participant-specific left and right anterior thalamic peak activation voxels (appendix e-1, doi.org/10.5061/dryad.2bvq83bm8).^32^ The PPI general linear model included 3 regressors: (1) main effect of the seed region (i.e., the functional time series), (2) task regressor (i.e., psychological factor, represented by the vector of the word-generation block onset), and (3) interaction of the former 2, representing a task-modulated change in connectivity, or PPI.^31^ Motion parameters were included as nuisance regressors. One-sample *t* tests identified areas exhibiting task-related connectivity changes with the thalamic seeds. Two-sample *t* tests compared TLE-FBTCS and TLE-FS groups, as well as left and right TLE subgroups. Main PPI effects were thresholded at *p* < 0.05, FWE-corrected across the whole brain. In view of our a priori hypotheses, group differences were considered significant at *p* < 0.05, FWE-corrected within a 12 mm-diameter sphere (FWE-svc) centered at the maxima in the hippocampus and motor areas.^27^ For completeness, whole-brain effects are reported at an exploratory statistical threshold of *p* < 0.005, k = 20.^28,29^

### Graph-theoretical analysis

Further image processing included regression of nuisance variables, bandpass filtering (0.01–0.1 Hz), and removal of the superimposed blocked task structure via condition-specific regressors, in line with benchmark evidence (appendix e-1, doi.org/10.5061/dryad.2bvq83bm8). Regional parcellation was attained via the Brainnetome atlas (246 ROI).^33^ After extracting ROI-averaged time series, we computed absolute Pearson correlation coefficients for every possible ROI pair, obtaining a 246 × 246 connectivity matrix for each participant. Weighted matrices were thresholded and binarized at network densities between 5% and 40% in increments of 1%, yielding 36 binary undirected graphs per participant. Bilateral thalamic parcels (regions 231/232, corresponding to a left/right anterior thalamic division) were identified as nodes for network statistics. We investigated measures of centrality (hubness), in light of their relevance for clinical outcome prediction in TLE.^9^ For each node at each network density, we computed (1) degree centrality, describing the number of connections of a given node, and (2) betweenness centrality, describing the frequency with which a given node is located on the shortest path between other node pairs. Differences in thalamic centrality between TLE-FBTCS and TLE-FS, and for left and right TLE subgroups, were assessed via (1) comparisons of mean metric values, obtained after averaging across densities,^34^ and followed up with (2) subsequent contrasts for each network density value for each metric. We used nonparametric permutation tests entailing 10,000 permutations for each comparison, which generated permuted *t* statistic distributions with associated *p* values,^9^ followed by FDR adjustment for multiple testing (*p*_FDR_ < 0.05; appendix e-1, doi.org/10.5061/dryad.2bvq83bm8).

### Receiver operating characteristic (ROC) curves with thalamic functional markers

ROC curves assessed the accuracy with which age- and sex-adjusted thalamic functional metrics could discriminate between TLE-FBTCS and TLE-FS. Initial models implemented markers of activation, extracted from the left ventral anterior thalamic parcel of the Morel atlas. To characterize the additional contribution of connectivity and graph metrics, ROC curve analyses were repeated using a composite functional construct, obtained after PCA on measures of activation, task-based connectivity, and centrality (appendix e-1, doi.org/10.5061/dryad.2bvq83bm8). Logistic regressions quantified the additive discriminative potential of activation and connectivity metrics. Models were compared via likelihood ratio tests.

### Post hoc analyses on 3 TLE groups

Post hoc analyses examined TLE with (current) FBTCS, TLE remote FBTCS, and TLE never FBTCS regarding parameter estimates of thalamic and hippocampal activation, thalamotemporal and thalamomotor task-related connectivity, degree and betweenness centrality. Across all analyses, we specifically tested the hypothesis that altered thalamic network embedding would relate to a current propensity for secondary generalization and, consequently, that there would be no significant differences between individuals with remote FBTCS and never FBTCS. Subgroups were compared via multivariate and univariate analysis of variance (ANOVA), along with nonparametric permutation ANOVA for graph-theoretical metrics. Extraction of activation and connectivity metrics and statistical procedures are detailed in appendix e-1 (doi.org/10.5061/dryad.2bvq83bm8)*.*

### Data availability

Data supporting our findings are available from the corresponding author upon reasonable request.

## Results

### Demographic and clinical characteristics

There were no differences between TLE-FS and TLE-FBTCS for demographic and clinical variables, including temporal pathology subtype, number of AEDs, and usage of topiramate or zonisamide, which both affect verbal fluency activations^28^ (all *p* > 0.05; table 1). Subgroup analyses, comparing LTLE-FBTCS against LTLE-FS, and RTLE-FBTCS against RTLE-FS, identified no significant differences. Propensity for FBTCS was similar in LTLE and RTLE subgroups (χ2 = 2.66, *p* = 0.10). A history of comorbid affective disorders was documented for 36.3% and 45.5% of patients with TLE-FS or TLE-FBTCS, respectively, with no group differences. Scores for anxiety and depression symptoms and usage of antidepressant/anxiolytic medication did not differ between groups (table 1 and appendix e-2, doi.org/10.5061/dryad.2bvq83bm8).

### Cognitive measures and volumetric findings

There were no differences between patients with TLE-FBTCS and patients with TLE-FS for all cognitive measures and thalamic and hippocampal volumes (all *p* > 0.05; table 1). Subgroup analyses detected a difference between LTLE-FBTCS and LTLE-FS regarding letter fluency scores, with LTLE-FBTCS outperforming LTLE-FS (*p*_FDR_ = 0.01). Consequently, sensitivity analyses addressed confounding effects of fluency performance on imaging metrics. Additional analyses indicated that differences in letter fluency between LTLE-FS and LTLE-FBTCS were largely mediated by hippocampal volume, processing speed, and medication, all of which had no influence on thalamic activation, connectivity, and graph-theoretical metrics (linear regression models, all variables *p* > 0.23; appendix e-2, doi.org/10.5061/dryad.2bvq83bm8). There were no other significant differences for cognitive and volumetric measures between LTLE and RTLE subgroups.

### Verbal fluency fMRI: activation-based analysis

**Figure 1:**
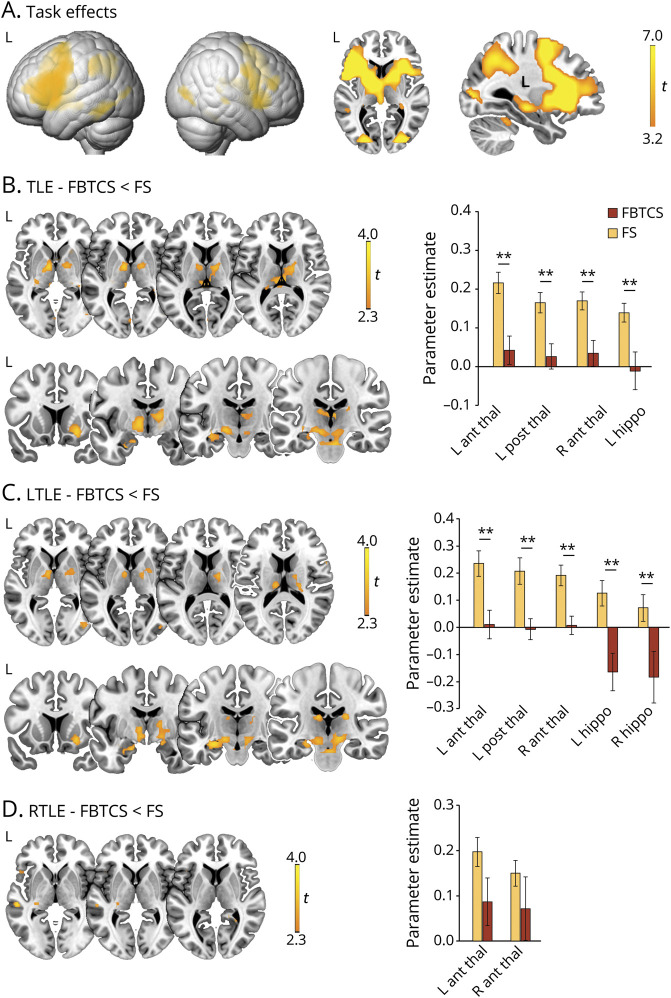
Verbal fluency fMRI activations (A) Whole-brain verbal fluency activations across all participants, as derived from 1-sample *t* tests. Axial and sagittal slices highlight activation of the thalamus, basal ganglia, and hippocampus. (B–D) Comparisons between temporal lobe epilepsy (TLE)–focal seizures (FS) and TLE–focal to bilateral tonic-clonic seizures (FBTCS) for task-related activation (B), and repeat contrasts for the same subgroups in left TLE (LTLE) (C) and right TLE (RTLE) (D). Axial slices specifically highlight differences in thalamic activation. Across B to D, bar graphs display statistical parametric mapping–derived parameter estimates of thalamic activation for areas of peak intergroup differences, namely left anterior/posterior thalamus, right anterior thalamus, and left hippocampus, for TLE-FBTCS vs TLE-FS (B); all the former plus right hippocampus for LTLE subgroups (C); and left/right anterior thalamus for RTLE subgroups (D); in the latter case, thalamic activation differences did not reach statistical significance, but bar graphs are reported for completeness. Rendered images in A are thresholded at *p* < 0.05, familywise error (FWE)–corrected for multiple comparisons across the whole brain. Across all panels, heat maps refer to brain slices, and display *t* scores. Montreal Neurological Institute coordinates and *p* values for group comparisons are provided in table 2 and table e-1 (doi.org/10.5061/dryad.2bvq83bm8). In bar graphs: **p < 0.05, FWE–small volume correction for peak intergroup difference.

The task elicited the expected^28^ activation of language-relevant fronto-temporo-parietal cortices, hippocampus, putamen, and pallidum with left-sided emphasis, as well as right cerebellum (figure 1A). Thalamic activation encompassed bilateral anterior divisions and left-sided posterior nuclei, with local maxima in the ventral anterior parcel of the Morel atlas.

Patients with TLE-FBTCS had less task-related activation of bilateral anterior and posterior thalamus and left anterior hippocampus than patients with TLE-FS (*p* < 0.05, FWE-svc; figure 1B and table 2). Peak thalamic activation differences fell within ventral anterior nuclei; additional peaks were detected in the centrolateral/lateral posterior group. Exploratory whole-brain analyses detected lower activation in TLE-FBTCS in bilateral posterior parahippocampal gyrus and subcortical structures including putamen, pallidum, cerebellum, and subthalamus (figure 1B, second row). Sensitivity analyses controlling for fluency performance did not affect anterior thalamic findings, but reduced significance of hippocampal and right posterior thalamic differences (table 2). There was no increased activation in TLE-FBTCS compared to TLE-FS.

**Table 2:**
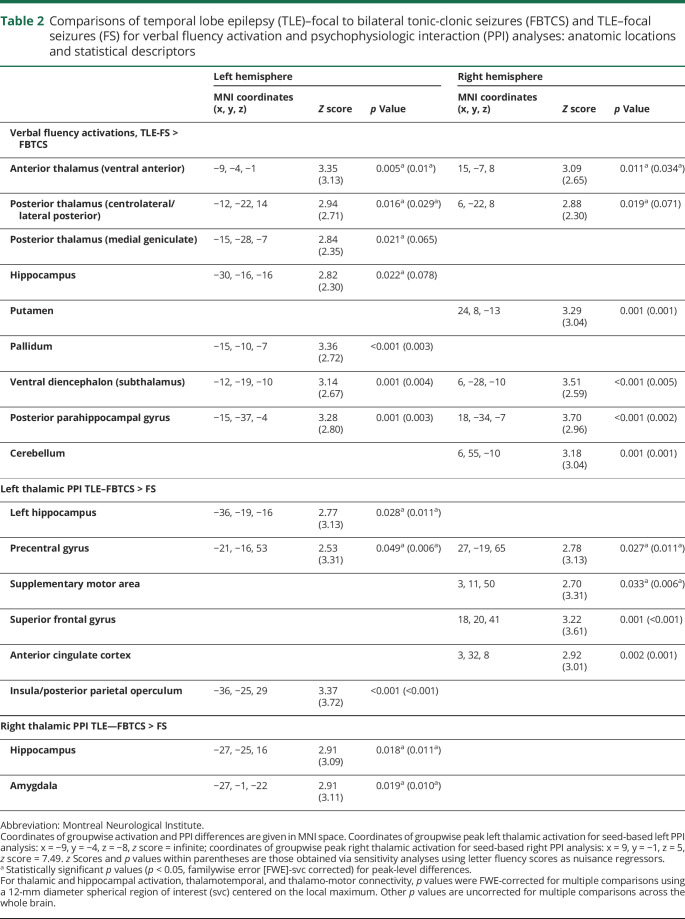
Comparisons of temporal lobe epilepsy (TLE)–focal to bilateral tonic-clonic seizures (FBTCS) and TLE–focal seizures (FS) for verbal fluency activation and psychophysiologic interaction (PPI) analyses: anatomic locations and statistical descriptors

Post hoc analyses contrasted TLE subgroups with a sample of 53 healthy controls, balanced for demographic variables (appendix e-1, doi.org/10.5061/dryad.2bvq83bm8). Thalamic activation was comparable to controls in TLE-FS, and significantly lower in TLE-FBTCS (all *p*_FDR_ < 0.0003), while hippocampal activation appeared reduced in both groups, with subtle effects in TLE-FS (*p* = 0.015, uncorrected; *p*_FDR_ = 0.075), and marked changes in TLE-FBTCS (*p*_FDR_ < 0.0001; figure e-1 and appendix e-2, doi.org/10.5061/dryad.2bvq83bm8).

Subgroup analyses detected reduced activation of bilateral anterior thalamus, left posterior thalamus, and bilateral hippocampus in LTLE-FBTCS compared to LTLE-FS (*p* < 0.05, FWE-svc; figure 1C and table e-1, doi.org/10.5061/dryad.2bvq83bm8). The subregional distribution of thalamic differences was similar to the main analysis, with ventral anterior maxima, and exploratory whole-brain comparisons in LTLE-FBTCS showed hypoactivation of the same widespread subcortical areas described for the main analysis. Repeat models controlling for fluency performance did not affect subgroup findings. In RTLE, thalamic differences between FBTCS and FS were not significant (figure 1D).

Collectively, our findings indicate thalamic and hippocampal hypoactivation on verbal fluency fMRI in TLE-FBTCS.

### Multiple regression analysis on activation metrics

Multiple regression based on the full predictor set was significant (*F*_9,93_ = 3.17, *p* = 0.002; multiple *R*^*2*^ = 0.23, adjusted *R*^*2*^ = 0.16). Occurrence of FBTCS in the last year was the most significant determinant of thalamic activation, and the association was negative (β = −0.17, 95% confidence interval [CI] [−0.28, −0.05], *t =* −2.90, *p* = 0.005). Handedness, sex, and side of epilepsy also had significant effects (βs = −0.19/−0.13/−0.11, 95% CI [−0.35, −0.04]/[−0.24, −0.01]/[−0.22, −0.005], *t* = −2.53/−2.24/−2.06, *p* = 0.013/0.027/0.041, respectively). Interaction terms (FBTCS*handedness, FBTCS*lateralization, FBTCS*sex) were nonsignificant (all *p* > 0.05).

### Psychophysiologic interaction analysis

**Figure 2:**
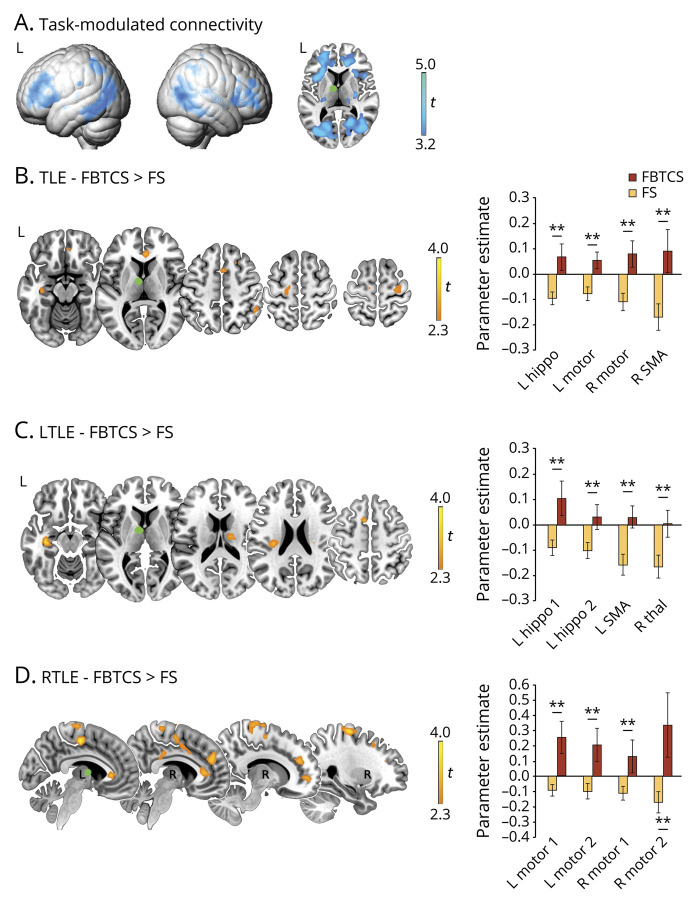
Psychophysiologic interaction (PPI) analysis: left thalamus (A) Task-modulated changes in left anterior thalamic connectivity across all participants. The green sphere in the axial slice corresponds to the left thalamic seed. (B–D) Comparisons between temporal lobe epilepsy (TLE)–focal to bilateral tonic-clonic seizures (FBTCS) and TLE–focal seizures (FS) (B), and repeat contrasts for the same subgroups in left TLE (LTLE) (C) and right TLE (RTLE) (D). Across B to D, bar graphs on the right display statistical parametric mapping (SPM)–derived parameter estimates of left thalamic PPI for areas of peak intergroup differences, namely left hippocampus, left/right precentral gyrus (motor cortex), and right supplementary motor area (SMA) for TLE-FBTCS vs TLE-FS (B); left hippocampus (2 spatially noncontiguous peaks), left SMA, and right medial dorsal thalamus for LTLE subgroups (C); left/right precentral gyrus (motor cortex; 2 spatially noncontiguous peaks on both sides) for RTLE subgroups (D). Rendered images in A are thresholded at *p* < 0.001, uncorrected for illustration purposes. Across all panels, heat maps refer to brain slices, and display *t* scores. Montreal Neurological Institute coordinates and *p* values for group comparisons are provided in table 2 and table e-1 (doi.org/10.5061/dryad.2bvq83bm8). In bar graphs: **p < 0.05, familywise error–small volume correction for peak between-group difference.

**Figure 3:**
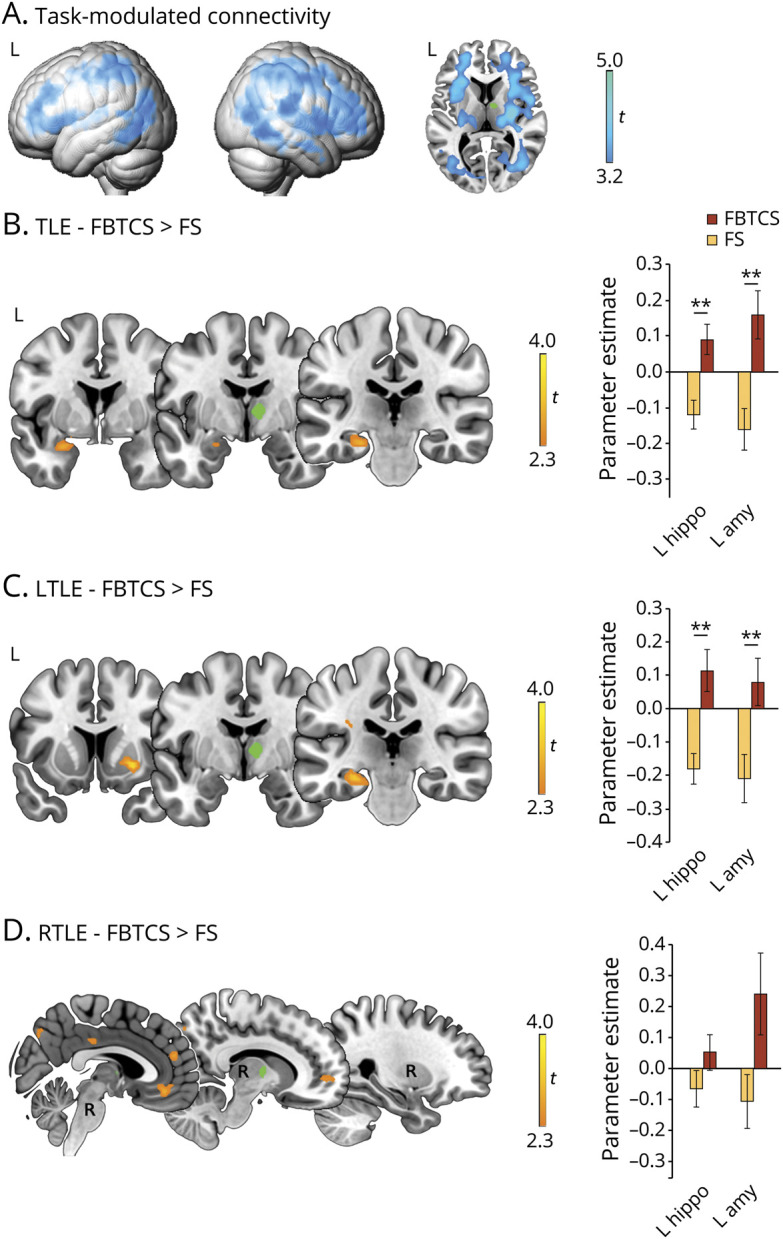
Psychophysiologic interaction (PPI) analysis: right thalamus (A) Task-modulated changes in right anterior thalamic connectivity across all participants. The green sphere in the axial slice shows the upper portion of the right thalamic seed. (B–D) Comparisons between temporal lobe epilepsy (TLE)–focal to bilateral tonic-clonic seizures (FBTCS) and TLE–focal seizures (FS) (B), and repeat contrasts for the same subgroups in left TLE (LTLE) (C) and right TLE (RTLE) (D). For B to D, bar graphs on the right display statistical parametric mapping (SPM)–derived parameter estimates of right thalamic PPI for areas of peak intergroup differences, corresponding to left hippocampus and left amygdala for all group comparisons. As for analyses in RTLE, bar graphs are reported for completeness, but group differences for hippocampal and amygdala activity did not reach statistical significance. Rendered images in A are thresholded at *p* < 0.001, uncorrected for illustration purposes. Across all panels, heat maps refer to brain slices, and display *t* scores. Montreal Neurological Institute coordinates and *p* values for group comparisons are provided in table 2 and table e-1 (doi.org/10.5061/dryad.2bvq83bm8). In bar graphs: **p < 0.05, familywise error–small volume correction for peak between-group difference.

PPI analysis showed task-modulated connectivity changes between the left thalamic ROI and fronto-temporo-parietal cortices, contralateral thalamus, basal ganglia, and mesiotemporal lobes (figure 2A). Overlapping effects were identified for PPI analysis from the right thalamus (figure 3A). In both cases, task-modulated changes in connectivity were negative, implying reduced thalamic functional connectivity (i.e., thalamocortical decoupling) as a function of task performance, in accord with previous evidence.^35^

Compared to TLE-FS, TLE-FBTCS exhibited less attenuated task-dependent connectivity (i.e., failure to reduce coupling) between left thalamus and both left hippocampus and motor areas, including bilateral precentral gyrus and right SMA (*p* < 0.05, FWE-svc; figure 2B and table 2). Additional whole-brain effects were detected in left posterior insula/operculum, right superior frontal, and anterior cingulate cortices. Stronger task-dependent left thalamic connectivity to left hippocampus, contralateral thalamus, and motor areas was observed in LTLE-FBTCS compared to LTLE-FS, whereas significant differences only encompassed thalamo-motor connections in RTLE-FBTCS vs FS (*p* < 0.05, FWE-svc; figure 2, C and D, and table e-1, doi.org/10.5061/dryad.2bvq83bm8). Controlling for verbal fluency performance increased statistical significance of all group comparisons (table 2 and table e-1, doi.org/10.5061/dryad.2bvq83bm8).

Similarly, PPI analyses from the right thalamus highlighted less attenuated connectivity to left hippocampus and amygdala in TLE-FBTCS compared to TLE-FS (*p* < 0.05, FWE-svc; figure 3B and table 2). Subgroup analyses showed higher connectivity to the left hippocampus in LTLE-FBTCS compared to LTLE-FS, and additional whole-brain effects were identified for the right putamen (figure 3C and table e-1, doi.org/10.5061/dryad.2bvq83bm8). In RTLE-FBTCS, stronger connectivity to left amygdala and right hippocampus was evident at uncorrected thresholds. Sensitivity analyses controlling for linguistic performance amplified the above-described effects.

Collectively, our results point to enhanced task-related thalamotemporal and thalamo-motor interactions in TLE-FBTCS.

### Graph-theoretical findings

**Figure 4:**
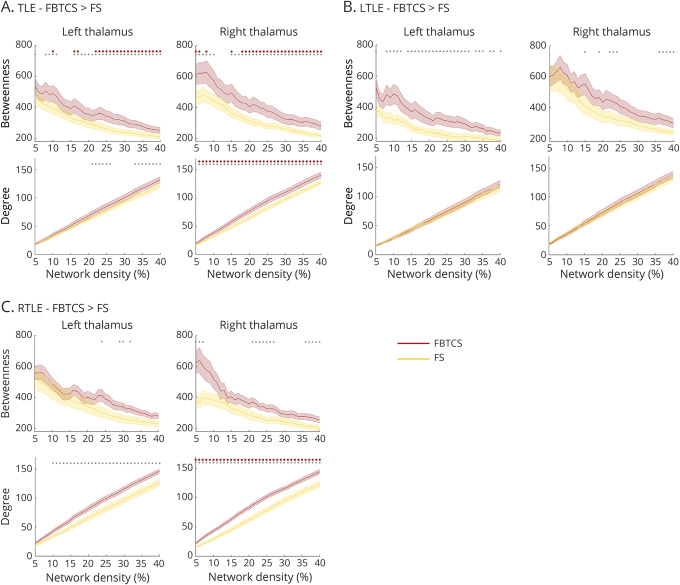
Graph-theoretical measures of centrality (A–C) Measures of betweenness and degree centrality of the left and right thalamic region of interest. Metrics for focal to bilateral tonic-clonic seizures (FBTCS) and focal seizures (FS) patient groups are displayed with dark red and orange lines, respectively. Shaded bands display standard errors, red dots indicate significant intergroup differences after false discovery rate correction for multiple comparisons (*p*_FDR_ < 0.05), gray dots indicate between-group differences at *p* < 0.05, uncorrected for multiple comparisons. Statistical details for comparisons of mean graph-theoretical metrics are provided in the main text. LTLE = left temporal lobe epilepsy; RTLE = right temporal lobe epilepsy; TLE = temporal lobe epilepsy.

TLE-FBTCS showed significantly higher mean betweenness centrality of bilateral thalamus and higher mean right degree compared to TLE-FS (uncorrected *p* = 0.037/0.033/0.032, respectively; all *p*_*FDR*_ = 0.049, adjusted across 4 measures). Differences for left degree were not significant (*p*_*FDR*_ = 0.10). Regarding the former significant measures, higher centrality in TLE-FBTCS was apparent across most network densities (figure 4). Subgroup analyses showed higher left thalamic betweenness centrality in LTLE-FBTCS compared to FS at the uncorrected level, both for mean values (*p* = 0.029 uncorrected, *p*_FDR_ = 0.12) and across network densities, and significantly higher right thalamic degree in RTLE-FBTCS vs RTLE-FS (*p* = 0.008 uncorrected, *p*_FDR_ = 0.030). Inspection of plots for the remaining nonsignificant comparisons showed overall trends for higher centrality in FBTCS subgroups.

### Individual discrimination via thalamic functional measures

ROC curve analyses based on left anterior thalamic activation discriminated between TLE-FBTCS and TLE-FS (area under the ROC curve [AUC] 0.67 [95% CI 0.56–0.77], *p* = 0.007). Subgroup analyses detected higher discrimination of LTLE subgroups (AUC 0.69 [0.55–0.83], *p* = 0.026), while findings in RTLE approached significance (AUC 0.67 [0.52–0.83], *p* = 0.06). Use of a composite functional marker, incorporating activation, task-related connectivity, and graph-theory metrics, achieved substantially higher discrimination than activation measures alone (ROC curve on combined metric, AUC 0.75 [0.64–0.85], *p* < 0.0001). Effects were more prominent for LTLE (AUC 0.83 [0.70–0.95], *p* = 0.0001), and also significant in RTLE (AUC 0.73 [0.58–0.89], *p* = 0.011). Comparison of logistic regressions via likelihood ratio tests (appendix e-1, doi.org/10.5061/dryad.2bvq83bm8) identified marked additive contributions of task-related connectivity to subgroup discrimination (*p* = 0.006), whereas addition of graph-theoretical metrics to the former 2 only yielded marginal improvements in model fit (*p* > 0.10).

### Post hoc analyses on 3 TLE groups

**Figure 5:**
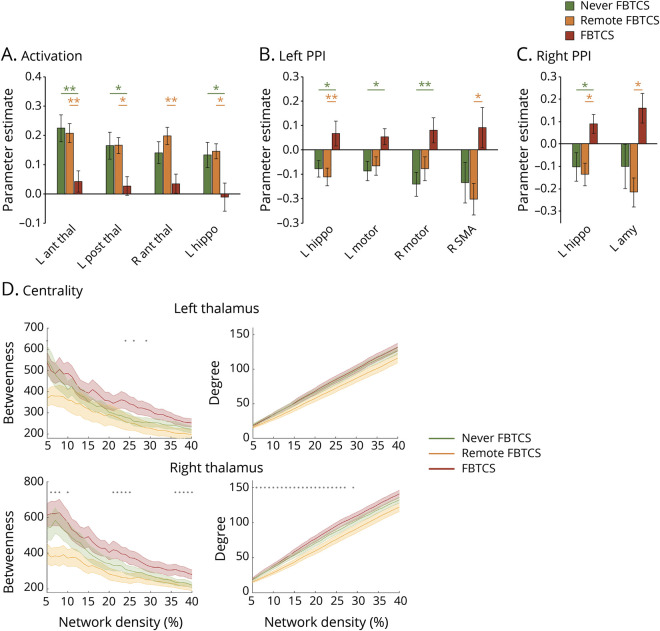
Post hoc analyses on 3 temporal lobe epilepsy (TLE) groups (A–D) Comparisons among (1) TLE with (current) focal to bilateral tonic-clonic seizures (FBTCS), corresponding to the group termed TLE-FBTCS throughout the article, (2) TLE remote FBTCS and (3) TLE never FBTCS (for further grouping details, see Methods). Bar graphs in A to C display parameter estimates extracted from locations of peak group differences in the main analysis on 2 groups, corresponding to: left anterior/posterior thalamus, right anterior thalamus, and left hippocampus for thalamic activation (A); left hippocampus, left/right precentral gyrus (motor cortex) and right supplementary motor area (SMA) for left thalamic psychophysiologic interaction (PPI) (B); left hippocampus and amygdala for right thalamic PPI (C). Montreal Neurological Institute coordinates of each location are provided in table 2 and e-1 (doi.org/10.5061/dryad.2bvq83bm8). (D) Group comparisons for measures of betweenness and degree centrality of the left and right thalamic region of interest. Shaded bands display standard errors, gray dots indicate between-group differences at *p* < 0.05, uncorrected for multiple comparisons. There were no significant intergroup differences after correction for multiple testing. In bar graphs: **p < 0.01, corrected (Tukey); *p < 0.05, corrected (Tukey).

Multivariate ANOVA (MANOVA) on measures of activation, left, and right PPI identified no significant differences between TLE never FBTCS and remote FBTCS (*p* = 0.25/0.60/0.63, respectively; *p* > 0.23 for all univariate analyses). MANOVA on 3 groups, on the other hand, confirmed significant effects for thalamic activity and, left and right PPI (*p* = 0.016/0.021/0.013, respectively; all *p*_FDR_ = 0.021), with corrected univariate post hoc analyses (Tukey range test) detecting differences for comparison of TLE-FBTCS vs either TLE never FBTCS or TLE remote FBTCS or both (figure 5 and table e-2, doi.org/10.5061/dryad.2bvq83bm8).

Analysis of thalamic graph-theoretical metrics via nonparametric ANOVA highlighted uncorrected group effects for bilateral betweenness centrality and right degree (figure 5). Post hoc tests indicated no statistically significant differences between TLE never FBTCS and TLE remote FBTCS for any metric at any network density level (all *p* > 0.05, uncorrected across network densities within each metric). Plot inspection confirmed the previously documented pattern of higher centrality in TLE-FBTCS. Separate analyses for LTLE/RTLE subgroups are described in figure e-2 and appendix e-2 (doi.org/10.5061/dryad.2bvq83bm8).

## Discussion

In TLE, previous research documented thalamic involvement during temporal lobe seizures^11,36^ and identified thalamic atrophy^5,37^ along with altered structural and functional connectivity.^38–40^ While much research focused on TLE as a whole, few investigations sought to identify markers of propensity for secondary generalization, and no studies investigated thalamic activation and connectivity during cognitive tasks. Using a verbal fluency fMRI paradigm, we document coexistence of attenuated thalamic and hippocampal activation with stronger task-modulated thalamotemporal connectivity and higher thalamic centrality in TLE with active FBTCS, compared to TLE with focal seizures only. Current presence of FBTCS was defined based on the occurrence of such seizures in the year preceding the investigation, in accordance with established clinical recommendations.^2,3,24^ Post hoc comparisons of patients with a history of remote FBTCS vs those with no lifetime experience of secondary generalization detected no significant differences in thalamic profiles, suggesting that the identified thalamic functional abnormalities specifically relate to the presence of active, uncontrolled FBTCS. By challenging a functional network largely overlapping with the putative epileptogenic network of TLE, our findings indicate impaired thalamic functional profiles as potential candidate markers of recurrent FBTCS, and thus disease severity.

Analysis of task-related activation detected reduced anterior and posterior thalamic recruitment in TLE-FBTCS compared to TLE-FS, with greater significance on the left. Hippocampal activation was also lower in TLE-FBTCS. Corroborating our a priori hypotheses, these findings indicate task-related disengagement of key components of the pathologic network of TLE in the subgroup with FBTCS, emphasizing the involvement of the thalamus, and advancing preliminary evidence of suboptimal hippocampal recruitment during language in TLE.^26^ From a neurobiological perspective, the fMRI signal relates to local field potentials, and likely reflects the extent of incoming input and local processes.^41^ Hence, we hypothesize that repeated insults of secondarily generalized epileptic activity may lead to more marked derangements of local neural activity and affect richness of synaptic connections, which may in turn explain impaired task-related recruitment of both hippocampus and thalamus in TLE-FBTCS. Discrepancies of effects emerging from the comparisons between left and right TLE subgroups may relate to task specifics, as verbal fluency fMRI paradigms implicate linguistic processing, and are particularly suited to capture effects within left hemispheric networks.^42^ Sensitivity analyses, including fluency scores as nuisance regressor, did not affect the results of the main group comparison and subgroup analyses, indicating that hippocampal and thalamic disengagement may occur during cognitive effort, but be independent of cognitive performance levels. We further confirmed subgroup comparability across a large series of clinical and demographic factors, including frontal and thalamic laterality indices. Moreover, multiple regression models identified FBTCS as the most significant determinant of anterior thalamic activation, among an extensive set of demographic, clinical, and cognitive measures.

Analysis of fMRI activation identifies areas implicated in task execution, but does not formally capture the interplay between those areas, known as functional connectivity. To assess thalamotemporal connectivity during task-based fMRI, we conducted a PPI analysis, providing measures of context-dependent, task-modulated changes in coupling between a seed region and the whole brain.^31^ PPI analysis from both left and right thalamus demonstrated attenuation of task-related connectivity to fronto-temporo-parietal cortices and subcortical targets, in accordance with previous results in healthy controls.^35^ Supporting a modulatory role of the thalamus during executive cognition, these findings relate to neurophysiologic studies indicating thalamus-driven synchronization and mediation of cortico-cortical information transfer.^43^ Group comparisons highlighted abnormal thalamotemporal interactions in TLE-FBTCS compared to FS, with less attenuated task-related connectivity between thalami and left hippocampus in the FBTCS subgroup, and altered connections between left thalamus and right anterior cingulate cortex. Stronger thalamotemporal coherence was particularly evident for comparisons of left TLE subgroups, while RTLE-FBTCS exhibited higher connectivity between thalamus and motor areas compared to RTLE-FS. Previous resting-state fMRI work in TLE documented bilaterally impaired connectivity of the posterior thalamus in TLE-FBTCS,^12^ but correlated thalamic time courses with those of cortical parcels with near-lobar extent. Here, we found that FBTCS relate to state-dependent connectivity differences affecting key components of the pathologic network of TLE, including limbic and rolandic areas. Task-based connectivity analysis thus provides an important complement to activation-based comparisons, by showing that reduced activation of hippocampus and thalamus is underpinned by stronger interregional synchrony and failure of reciprocal disengagement during cognition. From a mechanistic viewpoint, these findings may imply a reduced adaptability of neural communications within circuitry underlying secondary generalization, and highlight an association between recurrent FBTCS and more stereotyped, inflexible patterns of network interactions.

Graph-theoretical analysis allows tracking the organizational properties of brain networks, and centrality measures identify network hubs, i.e., regions with high connectivity to other network nodes and prominent influence over global network dynamics. In TLE, graph-theory investigations identified abnormalities of both mesiotemporal^44^ and whole-brain network architecture.^17^ Aberrant nodal topology was documented for limbic regions as well as thalamus,^45^ and recent work reported higher thalamic centrality as predictor of postsurgical seizure recurrence.^9^ Here, we identified higher anterior thalamic centrality in TLE-FBTCS compared to TLE-FS during a verbal fluency task, further supporting a relationship between FBTCS and higher thalamic functional integration within whole-brain networks. Our graph-theoretical results provide a third line of evidence for altered thalamic network embedding in TLE-FBTCS relative to TLE-FS. Higher centrality likely implies stronger connectional profiles and enhanced thalamic relevance within the context of whole-brain network architecture,^9^ which may underpin a network configuration facilitating diffuse dissemination of ictal discharges, and thus recurrent FBTCS.

To assess the potential clinical relevance of thalamic functional markers, we employed those within ROC curve analyses probing discrimination of TLE-FS and TLE-FBTCS. Although models already conveyed significant results with activation measures alone, discrimination abilities were substantially enhanced after combining measures of activity, connectivity, and centrality into a composite thalamic functional construct, reaching 75% accuracy for all TLE and >80% in LTLE. While proving the advantage of combining imaging metrics derived across investigative scales, these findings directly implicate thalamic functional profiles as potential surrogate marker of secondary generalization, with validity at the individual level.

Overall, our results dovetail with evidence from animal models, documenting the pivotal role of impaired thalamic gating for propagation and maintenance of seizures involving the neocortex,^46^ and the efficacy of thalamotomy in suppressing the latter.^47^ In patients with TLE, high-frequency thalamic stimulation desynchronizes hippocampal and large-scale epileptic network activity and induces cortico-cortical decoupling,^48^ which may underlie the efficacy of deep brain anterior thalamic stimulation.^49^ Our findings also complement recent resting-state fMRI evidence for abnormal interactions between thalamic divisions and basal ganglia in TLE with recent FBTCS.^50^ While differing methodologically, both analyses compellingly indicate a prominent role of the thalamus in shaping susceptibility to uncontrolled secondary generalization in TLE.

Our task-based fMRI investigation indicates reduced thalamic activation coupled with enhanced thalamotemporal connectivity and whole-brain thalamic network embedding as a functional signature of recurrent FBTCS in TLE. These patterns appear dynamic, and specifically relate to the presence of recent, uncontrolled secondary generalization. Altered thalamic network engagement is proposed as an imaging biomarker of active FBTCS, and thus disease severity, in TLE. While shedding light on the potential network correlates of recurrent FBTCS, our study delivers a viable target to track individual response to treatment and assess efficacy of novel therapeutic strategies directed toward generalization of focal seizures and SUDEP.

## Glossary

AED: antiepileptic drug
ANOVA: analysis of variance
AUC: area under the receiver operating characteristic curve
CI: confidence interval
FBTCS: focal to bilateral tonic-clonic seizures
FDR: false discovery rate
FS: focal seizures
FWE: familywise error
LTLE: left temporal lobe epilepsy
MANOVA: multivariate analysis of variance
NHNN: National Hospital for Neurology and Neurosurgery
PCA: principal component analysis
PPI: psychophysiologic interaction
ROC: receiver operating characteristic
ROI: region of interest
RTLE: right temporal lobe epilepsy
SMA: supplementary motor area
SUDEP: sudden unexpected death in epilepsy
svc: small volume correction
TLE: temporal lobe epilepsy
